# Webgroups: A way of facilitating web-based professional interaction - Initial experience at the IRIA, Delhi state branch

**DOI:** 10.4103/0971-3026.41842

**Published:** 2008-08

**Authors:** Ankur Dev, Lalendra Upreti, Sunil Puri, MK Mittal

**Affiliations:** GB Pant Hospital, JL Nehru Marg, New Delhi - 110 002, India. E-mail: lupreti@rediffmail.com; 1 Vardhman Mahavir Medical College and Safdarjung Hospital, Ring Road, New Delhi - 110 029, India

Dear Sir,

With the advent of the Internet, communication and teaching are gradually shifting from the print to the electronic media. The website of the Indian Journal of Radiology and Imaging (IJRI) receives 4000 hits daily.[1] In a study conducted among radiologists in the US, 97% of respondents indicated that they used the Web for education.[2] The setting up and maintenance of websites involves both initial and recurring monetary liabilities. Webgroups are an innovative tool for maintaining an online presence; they have the same basic functionality that full-fledged websites have but do not entail any significant financial burden.

**Figure 1:**
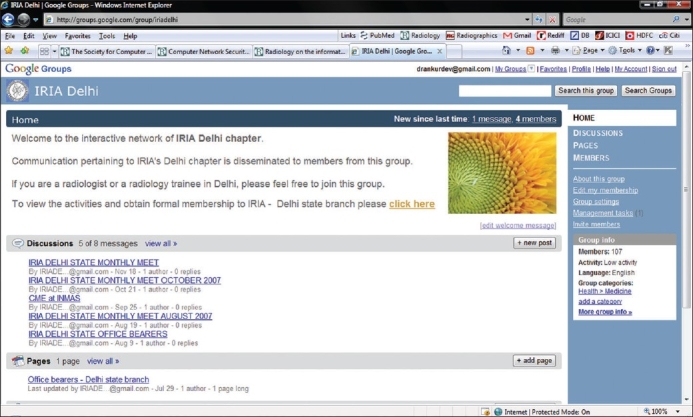
Screen capture of the webpage of the Delhi chapter of the IRIA

We have started a webgroup of the Indian Radiology and Imaging Association (IRIA) - Delhi state branch at the uniform resource locator (URL) http://groups.google.com/group/iriadelhi?lnk=gschg [Figure 1]. Two of the authors (AD and LU) have been chosen as moderators for administering the webgroup. The moderator's job is to approve members who sign up directly at the group's webpage, thus eliminating spam and misuse. All posted messages are screened before being displayed online. The moderators also post details of the monthly or annual meetings of the Delhi chapter of the IRIA and other CME programmes. These posts are emailed automatically to all members apart from being universally accessible at the group webpage.

Google groups is a free webgroup hosting service provided by Google Inc. Disseminating information through a webgroup is fast, entails lower costs than traditional postage, saves stationary, and is eco-friendly. The finances thus saved can be channelized into more productive avenues. Some disadvantages of using a group instead of a regular website include the inability to offer advanced functions such as payment gateways or an online voting system for state body elections.

Future directions include posting of interesting cases, online registration for conferences and CMEs, and uploading scientific sessions of CME programs.
